# Mineral Trioxide Aggregate Mixed with 5-Aminolevulinic Acid for the Photodynamic Antimicrobial Strategy in Hard Tissue Regeneration

**DOI:** 10.3390/ma11091734

**Published:** 2018-09-14

**Authors:** Yu-Fang Shen, Tsui-Hsien Huang, Hooi-Yee Ng, Hsin-Yuan Fang, Tuan-Ti Hsu

**Affiliations:** 1 Department of Bioinformatics and Medical Engineering, Asia University, Taichung City 40447, Taiwan; cherryuf@gmail.com; 2 3D Printing Medical Research Institute, Asia University, Taichung City 40447, Taiwan; 3 School of Dentistry, Chung Shan Medical University, Taichung City 40447, Taiwan; thh@csmu.edu.tw; 4 Department of Stomatology, Chung Shan Medical University Hospital, Taichung City 40447, Taiwan; 5 School of Medicine, China Medical University, Taichung City 40447, Taiwan; hooiyeen@gmail.com; 6 3D Printing Medical Research Center, China Medical University Hospital, Taichung City 40447, Taiwan; fanghy@mail.cmuh.org.tw; 7 Department of Thoracic Surgery, China Medical University Hospital, Taichung City 40447, Taiwan

**Keywords:** mineral trioxide aggregate, aminolevulinic acid, photodynamic therapy, dental pulp cell, odontogenesis

## Abstract

Aminolevulinic acid (ALA) based photodynamic antimicrobial strategy can provide good antimicrobial effects and be used for medical applications. The aim of this study was to apply this strategy to Mineral Trioxide Aggregate (MTA), which is commonly used as a filling material for root endings and by doing so, to increase the bactericidal capability of MTA, as well as to investigate its characterization, cytocompatibility, and odontogenic differentiation potential. MTA is known to be a derivative of calcium silicate (CS). In this study, MTA specimens with or without ALA and light treatment were prepared. Diametral tensile strength values (DTS), setting durations, X-ray diffraction (XRD) spectra, apatite-mineralization, and antimicrobial abilities of the MTA, were also analyzed. Human dental pulp cells (hDPCs) can proliferate into the newly formed matrix and differentiate into odontoblasts to reinforce and strengthen the root. Levels of hDPCs proliferation and its odontogenic capabilities when cultured on MTA with ALA and light treatment, and the percentages of cells existing in the various cell cycle stages, were further evaluated in this study. The results indicated that MTA added ALA with light treatment had greater antibacterial ability and cytocompatibility, compared to MTA alone. A higher percentage S phase of the cells cultured on MTA added ALA with light treatment was observed. Furthermore, hDPCs cultured on MTA added ALA with light treatment had the highest expression levels of the odontoblastic differentiation markers. ALA has great antimicrobial efficiency and is a potential material for future medical applications. ALA-based photodynamic antibacterial strategy applied in the MTA has great antibacterial ability, cytocompatibility, and odontoblastic differentiation potential, and can facilitate the development of root canal treatment.

## 1. Introduction

The occurrence of periapical disease is directly related to the presence of microorganisms in the dental pulp. The use of antimicrobial agents for the chemomechanical preparation of infected root canals, followed by obturation and coronal repair, provides beneficial results. However, sometimes infective microorganisms that reside in the root canals may gradually gain multiple resistance to drugs or disinfection methods, and this would eventually lead to the failure of root canal treatment [1,2] and more severe problems, such as chronic apical periodontitis and local bone resorption [3]. In such cases, endodontic surgery is commonly required to eradicate these microorganisms, and such surgical procedures usually include the mechanical removal of part of the infected tooth, followed by irrigation of antibacterial agents into the cavity before manual filling of cavities with impermeable body fluids, which have the ability to adhere to dentinal walls [4,5].

The efficiency of such disinfection is normally dependent on the antibacterial capability of the irrigation agents used, as well as the sealing ability of the sealers applied [1,2,6]. However, it has been constantly proven that classical irrigating agents are limited in eradicating microorganisms, especially in the root canals, due to the internal anatomical complexity of the root canals [7,8]. Often, there are residual bacteria post treatment and these bacteria can remain viable in dentinal tubules, and cause secondary re-infections of the root canals [8,9]. Therefore, there is a need for an alternative solution, which can increase efficiency of eliminating perilous microorganisms. To solve such problems, numerous studies have been conducted to develop novel endodontic sealers with sustained release capabilities of antibacterial substances to allow post eradication of residual bacteria, and to prevent secondary re-infection after post treatments [6,10].

Therefore, an ideal sealer for such dental surgical procedures should possess the following characteristics: It has to be biocompatible, should have optimal biological and physiological properties to facilitate tissue regeneration, should prevent microleakage, and concurrently be able to sustain the release of antibacterial substances to prevent re-infections [11,12,13]. As a calcium silicate-based (CS) material, mineral trioxide aggregate (MTA) shares similar chemical components as CS, and thus is also comprised of CaO, SiO_2_, Al_2_O_3_, and Bi_2_O_3_, and MTA has been reported to have an excellent sealing ability, anti-microleakage property, high biocompatibility and antibacterial activities, as well having anti-inflammatory properties and being a good odontogenic material [14,15,16]. In a previously reported study, it was reported that the components released by CS (i.e., Ca and Si ions), were the main culprit and mechanism behind the enhanced cellular behavior and antibacterial effects of CS. Furthermore, it was deduced that the antibacterial activity of CS might be due to the release of hydroxide ions (OH−) by CS, which caused pH values to be elevated [17,18]. Even though MTA was found to possess its own intrinsic antibacterial activity, it was reported that the activity levels of MTA were insufficient in conducting a complete eradication of perilous residual bacteria from the cavities. The final goal of root canal therapy is to achieve proper sealing of cavity with no secondary re-infection. Therefore, till today, many studies were being conducted to try to develop a sealer with superior antibacterial activity. For instance, Cetenovic et al. introduced chitosan nanoparticles, which possess excellent antibacterial characteristics, within MTA to up-regulate the bactericidal effects of MTA [19].

Recently, researchers had discovered a novel disinfection technique termed as photodynamic therapy (PDT). PDT works by irradiating a definite wavelength of light onto non-noxious photosensitizers and oxygen to bring about reactive oxygen species, specifically singlet oxygen, which was reported to be lethal towards bacteria [20]. In addition, it was reported to be less toxic against mammalian cells and has a higher antibacterial efficacy, compared to typical chemical irrigation solutions (i.e., sodium hypochlorite) [21]. More importantly, PDT was also reported to possess superior antibacterial ability against multi-resistant bacterial strains [22]. With reference to such contexts, PDT had recently been recommended as an alternative proposition to traditional endodontic strategies [23,24]. Porphyrin synthesis pathway is involved in mammals heme production and plants chlorophyll development, of which, an endogenous non-protein amino acid called 5-aminolevulinic acid (ALA) is the precursor for this pathway. In non-photosynthetic eukaryotes, such as animals, ALA is biosynthesized by the enzyme ALA synthase from its precursor compounds glycine and succinyl CoA. Specifically, for humans, 5-ALA exists as a precursor for heme. 5-ALA is formed in the cytosol and gets transported into the mitochondria, for further conversion to protoporphyrin IX. Following which, protoporohyrin molecules would then chelate with iron in the presence of an enzyme ferrochelatase to form heme. In macrophages, excess heme is converted into biliverdin and ferrous ions by an enzyme heme oxygenase-1 (HO-1). Furthermore, heme was reported to increase basal metabolic rate by elevating mitochondrial activity and activating the respiratory system (Krebs cycle and Electron transport chain), thus enhancing the formation of ATP. Therefore, 5-ALA is commonly used as a precursor molecule and as an agent for PDT due to its downstream above-mentioned genotoxic effects, which were reported to have enhanced DNA damage and methylation functions. These downstream properties are the reasons behind the excellent antibacterial properties of PDT [25]. Clinically, PDT is used as a theranostic tool for cancer due to its shielding effects on normal cells, and its high precision towards rapid proliferative cells [26]. In addition, evidence also implied that up-regulated differentiation of human dental pulp cells (hDPCs) into odontoblasts and protective effects against stress-induced cytotoxicity, were mainly due to the presence of heme and porphyrins. Both are known to enhance heme oxygenase-1 secretion, and heme oxygenase-1 is the crucial protein behind the above-mentioned effects [27,28].

For this study, we fabricated MTA-ALA composites by applying PDT on MTA. The physical and chemical properties of MTA-ALA composite were also analyzed and evaluated. In addition to evaluation of the antibacterial activity of our composites, we also evaluated the odontogenic differentiation potential of MTA-ALA after irradiation with red light. The results showed MTA-ALA with PDT had good antibacterial activity, cytocompatibility, and odontogenic differentiation potential. This study can provide a new strategy in the development of alternative root canal treatment.

## 2. Materials and Methods

### 2.1. Preparation of MTA/ALA Specimens

All the MTA specimens used were prepared according to the instructions from the manufacturer. Briefly, a powder/liquid ratio consisting of 0.3 mL/g of MTA powder (ProRoot MTA; Dentsply, Tulsa, OK, USA) and liquid were mixed to produce the cement. At the same time, deionized distilled water (ddH_2_O) was used to dissolve ALA (Sigma-Aldrich, St. Louis, MO, USA) to a working concentration of 3 mM. The MTA cements were then mixed with ddH_2_O (MH) or ALA (MA) and set into Teflon molds that were 6 mm in diameter and 2 mm in height. To mimic clinical use, all specimens were directly exposed to 635 ± 5 nm wavelength of red light after being filled into Teflon molds, followed by subsequent incubation at 37 °C and 100% relative humidity, for a period of 24 h to allow the cements to take shape. The lesion surface was exposed to a red-light intensity of 100 mW/cm^2^ with an area of 1 cm^2^ for 30 s, and at a height distance of 1.5 cm. Furthermore, prior to in vitro studies, sterilization was done for all specimens by immersing them in 75% ethanol, and a subsequent 30 min ultraviolet (UV) irradiation [29]. A minimum of at 12 specimens were tested for this study.

### 2.2. Setting Time and Mechanical Properties

To test for the final setting timings of cements, 456.6 g Gilmore needles was used and the tests were conducted in accordance to the guidelines dictated in ISO 9917-1. In brief, the setting duration was determined to be at the moment when the needles were not able to indent 1mm into three separate areas of the specimens. EZ-Test (Shimadzu, Kyoto, Japan) was used to analyze for the diametral tensile strength with a constant crosshead speed of 1 mm min^−1^ and this test were done by in accordance to the ASTM D5024-95a guidelines. A minimum of at 12 specimens were tested for this study.

### 2.3. Phase Composition and Morphology

X-ray diffraction (XRD; Shimadzu XD-D1, Kyoto, Japan), in a context of over 2-theta range from 20° to 50° and a 1°/min scanning speed, was used to study for the crystalline phases of MTA-ALA, after the specimens were treated with red-light. In addition, scanning electron microscopy (SEM; JSM-6700F, JEOL, Tokyo, Japan) was applied to observe for cell morphologies on the MTA-ALA cements, afore and post, simulated body fluid (SBF) soaking.

### 2.4. In Vitro Soaking

Based on the assumption that crystallization of a hydroxyapatite layer on the surfaces of scaffolds was a requirement for inducing material-bones bonding, such formation of an apatite layer was commonly used as an indicator for level of bioactivity of materials, which was otherwise known as the ability of materials to bond with live bones. Therefore, to gauge for bioactivity, the specimens were soaked in 10 mL SBF for a day at the temperature of 37 °C. The SBF used in this study had similar ionic compositions as blood plasma of humans, which is made up of reagent graded 0.2235 g of KCl, 7.9949 g of NaCl, 0.147 g of K_2_HPO_4_, 0.3528 g of NaHCO_3_, 0.305 g of MgCl_2_·6H_2_O, 0.2775 g of CaCl_2_, and 0.071 of g Na_2_SO_4_, mixed evenly with 1000 mL of distilled H_2_O. The solution was then adjusted to pH 7.4 with trishydroxymethyl aminomethane (Tris, CH_2_OH)_3_CNH_2_) and hydrochloric acid (HCl). SEM was used to observe for the microstructures of the in vitro apatite crystallization on the specimens after one day of immersion.

### 2.5. Antibacterial Properties

*Staphylococcus aureus* (*S. aureus*) and *Pseudomonas aeruginosa* (*P. aeruginosa*) were employed to investigate the bactericidal effects of MTA-ALA, after undergoing 15 min of red-light treatment. In brief, after the irradiation, all specimens were cultured with both types of bacteria (at a concentration of 4.0 × 10^4^ bacteria per mL) in Lysogeny broth (LB, Sigma-Aldrich) culture medium for 24 h. After which, 0.1 mL of culture medium was extracted and mixed with 0.9 mL of PrestoBlue^®^ (ThermoFisher Scientific, Waltham, MA, USA) and incubated for 10 min, before transferring it into a 96-well plate for reading of absorbance. The absorbance was measured by a multi-well spectrophotometer (Infinite Pro M200, Tecan Austria Gesellscha, Salzburg, Austria), set at 570 nm wavelength, with a reference of 600 nm wavelength. Cells cultivated with normal medium and ALA-contained medium (Ctl and CtlA respectively) were used as controls for this study. Triplicate results from three separate tests were done, and results were presented in optical density (OD). At the same time, an agar diffusion test was conducted to test for susceptibility to antibiotics, and to test for the inhibitory effects of MTA-ALA on various bacterial strains. Agar diffusion test is thought to be a standard method for testing of susceptibilities. For this test, six sterilized disk-shaped samples of 6 mm each were placed on *Staphylococcus aureus* grown agar plates and incubated for a day. After which, the inhibition zones surrounding the disks were measured and presented in terms of mm.

### 2.6. Cell Isolation and Culture

Primary human dental pulp cells (hDPCs) were used to study for the interactions between materials and seeded cells. hDPCs were extracted from the extracted teeth of patients, and such extraction of cells were conducted in accordance to methods previously described in References [30,31]. Approval was sought from the Chung Shan Medicine University Hospital Ethics Committee (CSMUH No. CS14117), and informed consent was provided and collected from the patients. The premolars used for this study were extracted for initial orthodontic purposes and were further ensured to be caries-free and intact before proceeding with the extraction of cells. In brief, the tooth was sagittally cleaved using a chisel, followed by soaking of the pulp tissues in PBS (Caisson, North Logan, UT, USA) solution with 0.1% collagenase type I (Sigma-Aldrich) for 30 min to allow digestion, and then placed onto a new plate for subsequent culture with Dulbecco’s modified Eagle medium (DMEM; Caisson) in a humidified incubator set at 5% CO_2_ and 37 °C. The DMEM used for the initial culture was enhanced with 1% penicillin (10,000 UI/mL)/streptomycin (10,000 mg/mL) (PS, Caisson) and 20% fetal bovine serum (FBS; GeneDireX, Taipei, Taiwan), with replacement of the medium every 72 h. The cells were sub-cultured till passages 3-7, where they are deemed to be suitable for in vitro studies. The DMEM used for subsequent odontogenic differentiation was enhanced with 0.05 g/L l-Ascorbic acid (Sigma-Aldrich), 2.16 g/L glycerol 2-phosphate disodium salt hydrate (Sigma-Aldrich) and with 10^−8^ M dexamethasone (Sigma-Aldrich).

### 2.7. Cell Viability

hDPCs were used to study for relationships between MTA-ALA and cell viability. A density of 10^4^ cells/well of hDPCs were seeded on the specimens for 12 h. Following which, the specimens were removed from the wells and treated with red-light at an intensity of 2 J/cm^2^. After various durations of culture, PrestoBlue^®^ (ThermoFisher Scientific), known as a cell viability assay, which works by detecting the amount of mitochondrial activity, was applied to analyze for levels of cell proliferation. Briefly, 1:9 ratio of PrestoBlue^®^ solution to DMEM were mixed, pipetted into each well, and incubated for 45 min in an incubator. After which, 100 μL was removed and placed in a 96-well ELISA plate for reading of absorbance. The absorbance was measured by an Infinite Pro M200, set at 570 nm wavelength, with a reference of 600 nm wavelength. Cells cultivated with normal medium and ALA-contained medium (Ctl and CtlA respectively) were used as controls for this study. Triplicate results from three separate tests were done, and results were presented in optical density (OD).

### 2.8. Fluorescent Staining

After cell seeding and 24 h of culture on MTA-ALA specimens, DAPI (Invitrogen, Carlsbad, CA, USA) stain and F-actin stain were used to observe for the nuclei and cytoskeleton of hDPCs. In brief, cold PBS was used to rinse the cells, followed by subsequent fixation of cells with 4% paraformaldehyde (Sigma-Aldrich), and next, the cells were permeabilized with 0.1% Triton X-100 (Sigma) in PBS. For fixation, it was done at room temperature for 20 min. After which, Alexa Fluor 594-phalloidin (Invitrogen) was used to stain the cytoskeleton filaments for 2 h, and 300 nM of DAPI (Invitrogen) was used to stain the nuclei for 30 min. The stains were then raised with PBS, and a Zeiss Axioskop2 microscope (Carl Zeiss, Thornwood, NY, USA) was used to observe and capture images of morphologies of hDPCs.

### 2.9. Cell Cycle

After 24 h of cell culture, both suspended and adhered cells were accumulated, underwent centrifugation, and fixation with chilled 99% ethanol for 3 h, at −20 °C. PBS with 0.1% Triton X-100, 200 μg/mL of RNase A (Sigma-Aldrich) and 100 μg/mL of propidium iodide (PI, Invitrogen) was used to stain the cell suspensions for 2 h, at 4 °C with light protection. Flow cytometry (Becton Dickinson, Franklin Lakes, NJ, USA) was used to analyze cell quantities, and WinMDI 2.8 software (Scripps Research Institute, La Jolla, CA, USA) was used to analyze the quantity of cells existing in different cell cycle phases. Triplicate results from three separate tests with 10^4^ cells were done, and results were presented as the average of the three.

### 2.10. Western Blot

Cell lysates were used for the study of protein expressions, with the aid of Western blot analysis. The cell lysates were from hDPCs after seven days of culture. Briefly, hDPCs were lysed using NP-40 lysis buffer (Invitrogen) by incubating for 30 min at 4 °C. After which, the medium was centrifuged to obtain the lysates (40 μg protein), and further segregated using SDS-polyacrylamide gel electrophoresis (SDS-PAGE), before transferring to nitrocellulose membranes. Then, 5% bovine serum albumin (BSA, Gibco) was used to fix the proteins for 1 h and immunoblotted for 2 h with the following primary antibodies: Anti-dentin sialophosphoprotein (DSPP, sc-73632, Santa Cruz), β-actin (GeneTex, GTX109639, San Antonio, TX, USA) and Anti-dentin matrix protein-1 (DMP-1, sc-73633, Santa Cruz Biotechnology, Santa Cruz, CA, USA). The membranes were then washed thrice with tris-buffer saline containing 0.05% Tween-20 (Sigma-Aldrich). Further addition with horseradish peroxidase (HRP)-conjugated secondary antibody was done to visualize the protein bands with enhanced chemiluminescent detection kits (Invitrogen). A densitometer (Syngene Bioimaging System; Frederick, MD, USA) was used to scan the stained protein bands, and Image J 1.45 software (National Institutes of Health, Bethesda, MD, USA) was used to quantify the stained bands. For analysis and comparison, the values of protein expressions were all normalized to β-actin. Triplicate results from three separate tests were done for this study.

### 2.11. Osteogenesis Assay

Levels of alkaline phosphatase (ALP) activity and osteocalcin (OC) proteins were obtained from cells that were cultured for seven days and 14 days, respectively, to dictate the relationships between specimens and osteogenesis abilities. In brief, cells were rinsed with cold PBS, lysed using 0.2% NP-40, and underwent centrifugation for 10 min. P-nitrophenyl phosphate (pNPP, Sigma) was used to determine for ALP activities by adding pNPP mixed with 1 M diethanolamine buffer (equivalent ratio) for 15 min. Then, 5 N NaOH (Showa Chem. Ind. Co., Ltd., Tokyo, Japan) was used to stop the above reaction, followed by quantification of ALP by measurement of its absorbance. At the same time, BCA protein assay kit (Bio-Rad Laboratories, CA, USA) was used to determine the total protein concentration. For this study, the level of ALP activity was quantified by normalizing ALP levels to total protein concentrations. Triplicate results from three separate tests were done for this study.

For OC proteins, an osteocalcin enzyme-linked immunosorbent assay kit (#ab195214, Abcam, Cambridge, MA, USA) was used according to the instructions from the manufacturers, to quantify the amount of OC proteins. For this study, a standard curve was used to correlate the absorbances of level of OC proteins. Blank cartridges were used as controls, and triplicate results from three separate tests were done for this study.

### 2.12. Alizarin Red S Stain

Calcium depositions after seven and 14 days of cell culture were analyzed using Alizarin Red S staining in accordance to methods previously described in Reference [13]. In brief, the cells were fixed for 15 min using 4% paraformadedyde (Sigma-Aldrich), and further cultured using 0.5% Alizarin Red S (Sigma-Aldrich) for 15 min at pH 4.0 room temperature, whilst undergoing 25 rpm of oscillation. Cells were subsequently washed thoroughly, and an optical microscope (BH2-UMA, Olympus, Tokyo, Japan) furnished with a digital camera (Nikon, Tokyo, Japan, 200X) was used to capture images of the stains. Furthermore, the specimens were soaked in 1.5 mL of 5% SDS in 0.5N HCl at room temperature for 30 min, then centrifuged at 5000 rpm for 10 min. The supernatant was transferred to a 96-well plate to measure for the absorbance at 405 nm wavelength (Infinite Pro M200). The above protocol was done to quantify for the amount of calcium depositions after the Alizarin Red S stains.

### 2.13. Statistical Analyses

For consistency of data collection, data were gathered by a single observer, and statistical analysis of the studies were expressed as mean ± standard deviation (SD). A one-way analysis of variance with SAS 9.4 (SAS Institute Inc., Cary, NC, USA) was used to conduct inter-groups comparisons, and Scheffe’s multiple comparison test was used to conduct multiple comparisons. For this study, *p* < 0.05 was the determined statistical significance for all results presented.

## 3. Results

### 3.1. Physicochemical Properties

**Figure 1:**
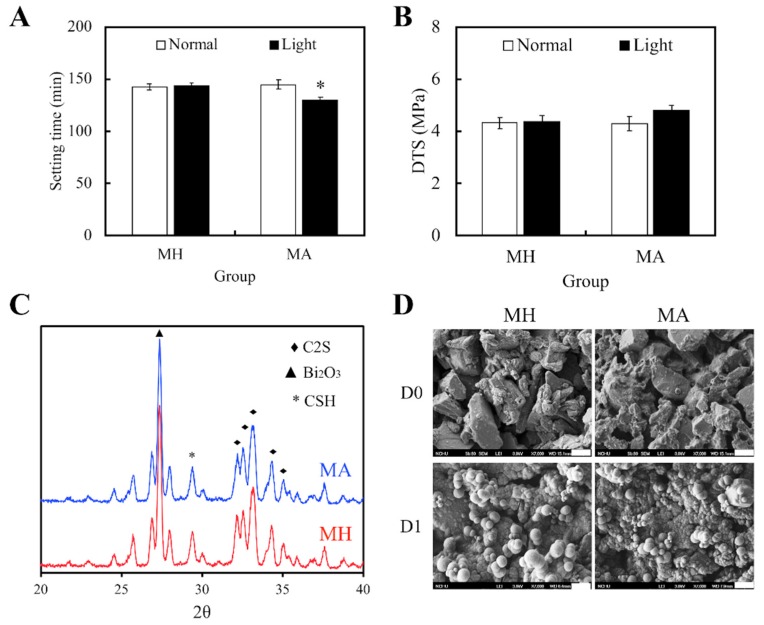
(**A**) Setting durations and (**B**) diametral tensile strength of Mineral Trioxide Aggregate (MTA) cements after Aminolevulinic acid (ALA) augmentation and light treatment. Statistical data are displayed as means ± standard deviations for *n* = 6. “*”, and the results exhibit statistically great differences (*p* < 0.05) in comparison to non-red light treated MTA. (**C**) X-ray diffraction (XRD) of MTA, with and without ALA, after a day of hydration at 37 °C. (**D**) SEM images of the surface of MTA-ALA scaffolds on day 0 and day 1 of simulated body fluid (SBF) immersion.

Figure 1 exhibits the physicochemical characteristics of our specimens, namely the MTA (MH), and both light- and non-light treated MTA-ALA (MA). The results suggested that treatment of light would not affect setting times and value of DTS for the ALA group. However, light treated MA had significantly reduced setting durations (*p* < 0.05), with slightly superior mechanical strength (*p* > 0.05), compared with the others (Figure 1A,B). With reference to the XRD patterns (Figure 1C), there was the presence of diffraction peaks at 2θ = 29.4°, which coincided with the presence of calcium silicate hydrate (CSH) ge; and diffraction peaks at 2θ between 32°–34°, which corresponded to the presence of β-dicalcium silicate (β-Ca_2_SiO_4_) residues. Therefore, from these results, it could be observed that there was an incomplete reaction with water. Furthermore, MA had mutually identical phase compositions with MH. SEM images (Figure 1D) of MA and MH after 1 day of immersion in SBF showed the formation of tiny spherical crystals and an apatite layer, which was indicated by the presence of small and large layered precipitates on the surface. These results revealed that the presence of ALA in MTA could also facilitate the formation of apatite spherule aggregates precipitation.

### 3.2. Antibacterial Properties

**Figure 2:**
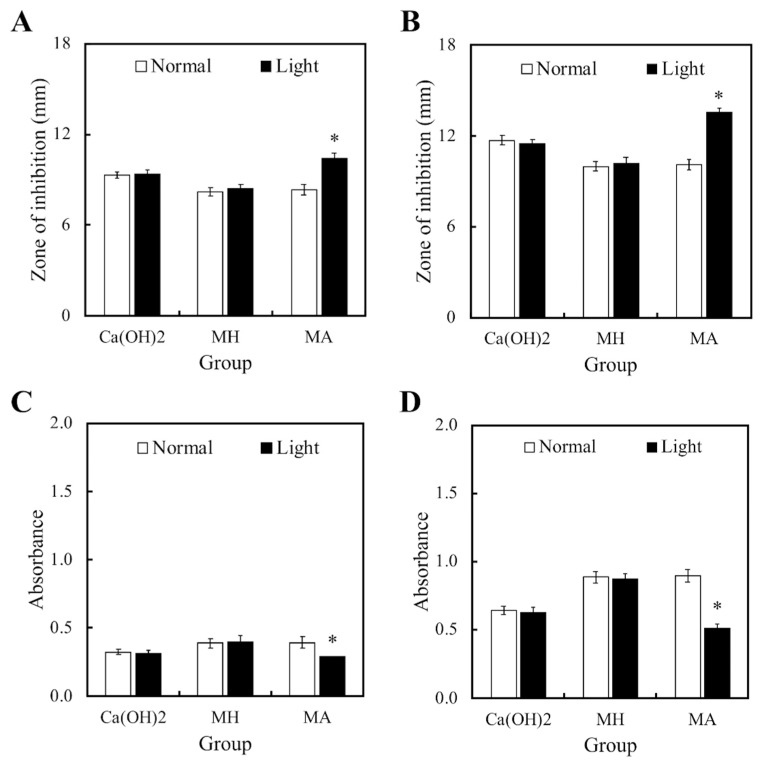
(**A**,**B**) Zones of inhibition and (**C**,**D**) the anti-bacterial effects of light- and non-light treated MTA-ALA on (**A**,**C**) *S. aureus* and (**B**,**D**) *P. aeruginosa*. Data are displayed as means ± SD (*n* = 6). “*”, with statistically significant difference (*p* < 0.05), compared to the substrate without red light irradiation.

Figure 2 displayed the bactericidal properties of both light and non-light treated MTA cements. Figure 2A,B showed the size of the inhibition zones in the cements cultured with *S. aureus* and *P. aeruginosa*, respectively. However, MTA had limited bactericidal capacity on both strains in comparison to Ca(OH)_2_. In addition, it was observed that MH and MA without light treatment had no effect on the antibacterial activity of MTA cements. On the other hand, bacteria growth in both strains were significantly inhibited in the MA with light treatment group. Figure 2C,D displayed identical trends with the inhibition zone results. These results demonstrated that MTA-ALA with light treatment had better antibacterial properties, compared to the rest of the groups.

### 3.3. Cell viability

**Figure 3:**
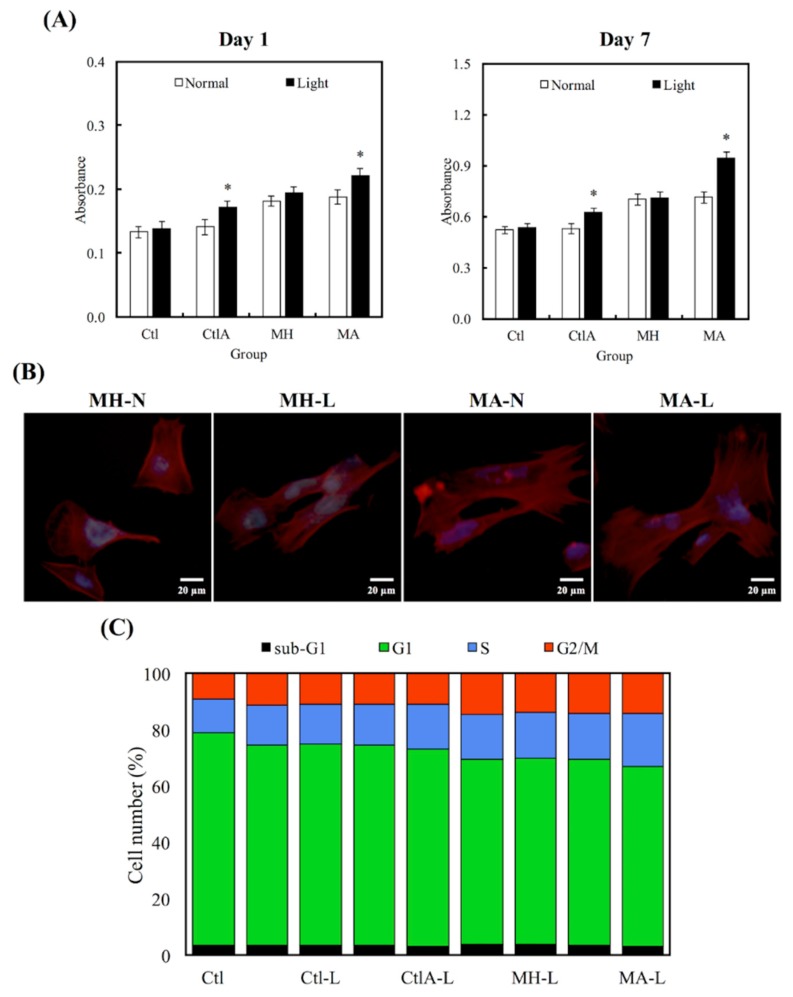
(**A**) Human dental pulp cells (hDPCs) proliferation assay cultured on different specimens at days 1 and 7. “*”, with a statistically significant difference (*p* < 0.05) from Ctl. (**B**) DAPI immunofluorescence images of cell nucleus (blue) and F-actin (red) of hDPCs on non-light treated MTA cements (MH-N and MA-N) and on light-treated MTA cements (MH-L and MA-L) after 1 day of culture. (**C**) The percentages of each cell cycle phase after a day of culture on Ctl, light-treated Ctl with normal medium and ALA-contained medium (Ctl-L and CtlA-L) and light-treated MTA groups (MH-L and MA-L), determined using flow cytometry.

Figure 3 showed the differences in cell proliferation of hDPCs on various substrates after 1 and 7 days, respectively. It is worthy to note that the viability of cells seeded on MA was much higher, compared to the viability of control (Ctl) groups throughout the course of culture. In addition, viability of hDPCs was not affected by the presence of ALA regardless of other external factors, such as difference in culture medium (Ctl) or presence of cement in MA (*p* < 0.05). Higher hDPCs viability for days 1 and 7 were constantly noted in the light treatment group, which can be observed in the ALA group, and especially more significantly in the MA group (*p* < 0.05). Figure 3B showed the fluorescence F-actin and DAPI nuclei stained images of hDPCs, and it can be clearly seen that MA-L had homogenous cell spreading and cells were well-spread with obvious cellular extensions, compared to the others. Figure 3C showed the percentages of cells existing at different cell cycle stages. Cells existing in the different phases of the cell cycle (G0, G1, S, and M phase) were presented as percentages of the total cells, and Ctl at the 0 h time point was 4%, 75%, 12%, and 9%, respectively. It was conspicuous that hDPCs had underwent G1 phase, S phase, and M phase, after 24 h of culture. The differences in percentages of different cell cycle phases were insignificant, when compared to all the controls (*p* > 0.05). In contrast with the control groups, cells seeded on MTA, disregarding whether it was treated with ALA or light, displayed a superior percentage of hDPCs exiting in the M stage. Light treated MA had the highest degree of cellular mitosis and duplication.

### 3.4. Odontogenic Differentiation in hDPCs

**Figure 4:**
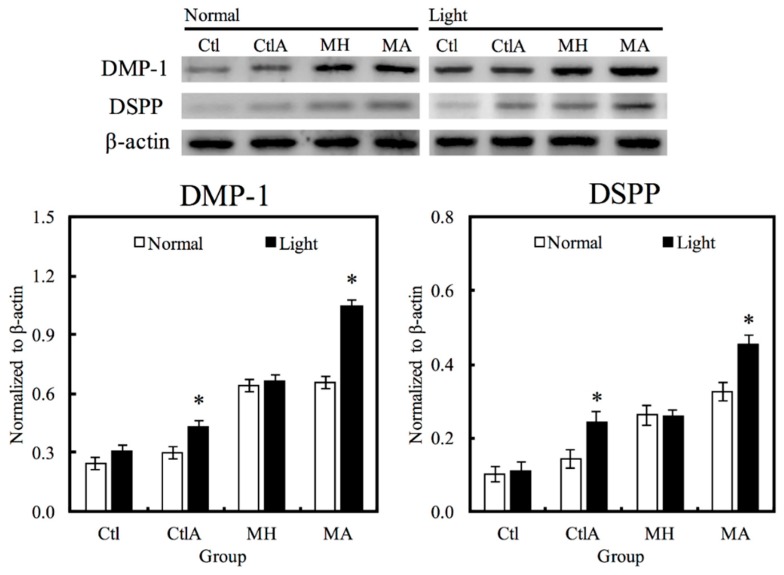
Western blot analysis of hDPCs protein expression (DSPP and DMP-1) cultured on MTA-ALA after 7 days of culture with red light treatment “*”, with a significant difference (*p* < 0.05) from specimens without red light treatment.

**Figure 5:**
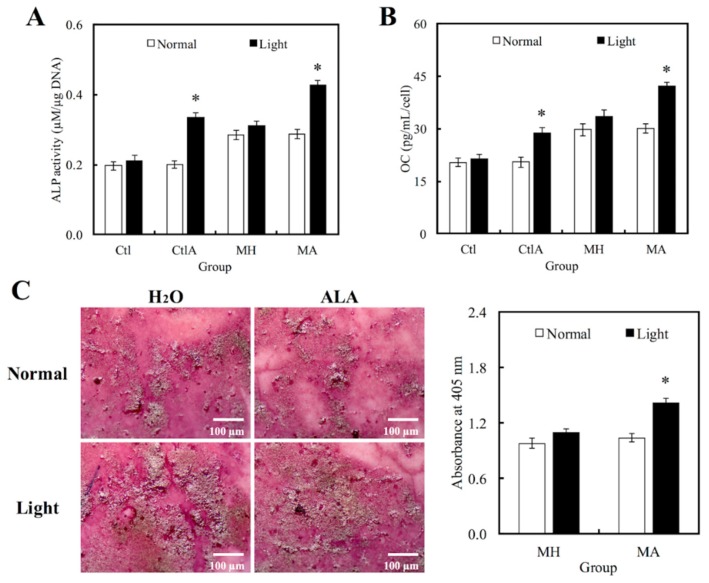
Levels of (**A**) Alkaline phosphatase (ALP) activity and (**B**) Osteocalcin (OC) secretion of hDPCs cultured on MTA-ALA after 7days of culture with red light treatment. (**C**) Images and quantification results of alizarin staining of calcium deposits after two weeks of culture. “*”, significant difference (*p* < 0.05) from specimens without red light irradiation.

Western blot analysis was used to evaluate hDPCs odontogenic differentiation ability in different culture conditions by studying the differentiation markers DSPP and DMP-1 (Figure 4). Compared to the controls, cells on MTA cements displayed significantly superior expressions of DSPP and DMP-1 (*p* < 0.05). Negligible hDPCs differentiation were observed in Ctl and MTA groups, regardless of whether there was presence of light or ALA treatment. However, a significant synergistic interaction was observed between light treatment and ALA in enhancing odontogenic differentiation of hDPCs (*p* < 0.05), as well as increasing ALP activity and OC secretion, respectively, as shown in Figure 5A,B. Highest ALP activity and OC level were observed in light-treated MA amongst all groups (*p* < 0.05). Alizarin Red S staining was used to stain for the presence of calcified deposits on the different scaffolds, as displayed in Figure 5C. From the results, it showed that MTA with light treated ALA had positive effects in facilitating cell formation by the increasing capacity of calcium nodule (Figure 5C). Calcium mineral deposit levels by hDPCs cultured on MA-light at culture periods of 14 days, were observed to be 1.32, 1.29, and 1.51 times greater than that on MA-normal, MH-light, and MH-normal (*p* < 0.05), respectively, according to the quantitative analysis. These results implied that the light-treated MTA-ALA might be able to activate the hDPCs mineralization.

## 4. Discussion

There are countless microorganisms in the oral flora of a normal healthy human being, and bacterial infection has always been a major complication of root canal therapy. In a previous study, Asnaashari et al. suggested that PDT can inactivate endodontic pathogens without affecting the survival of the host tissue, and PDT is a safe and effective method in clinical application [32]. Therefore, ALA based photodynamic antimicrobial strategy was applied on MTA to assess for its potential application in root-end filling material in this study. Its material characterization, cell viability, antibacterial, and odontogenic differentiation abilities were investigated. Previous studies showed that bacteria, fungi, yeasts, and some parasites can take up exogenous 5-ALA directly, which induced the accumulation of protoporphyrin IX (PpIX). PpIX was reported to have interactions with large sized molecules (i.e., calf thymus DNA) and DNA to bring about hyperchromic effects. In addition, PpIx was found to generate reactive oxygen species (ROS)to cause significant amounts of cell damage [33]. Although many different photosensitizing compounds, including methylene blue (7-(dimethylamino)-*N*,*N*-dimethyl-3Hphenothiazin-3-iminium chloride, MB), synthetic porphyrins, rose bengal (4,5,6,7-tetrachloro-2′,4′,5′,7′-tetraiodofluorescein disodium salt), and acridine (2,3-benzoquinoline) are known to be effective singlet oxygen generators, the useful photosensitizers are based on the structure of tetrapyrrole chromophore, such as porphyrin, bacteriochlorin (BC), and chlorin derivatives [34]. Therefore, ALA and its photodynamic antimicrobial characteristics is a promising strategy, which is shown to have better antimicrobial efficiency and is a potential material for future medical applications [19,35,36,37,38].

MTA is a popular endodontic material, which is commonly used as a filling material for root endings, pulp capping material, and also for perforation repair [14,39,40]. Despite having many advantages, its long setting timings had severely limited its use for clinical applications [41]. An alternative solution was proposed to eliminate this disadvantage, which is to combine MTA with light-treated ALA. The setting timings were significantly shorter when MTA was used in combination with light-treated ALA, compared to only MTA. Even though the setting timing was still longer than the optimal setting timing of 10–15 min [17], there was a slight improvement through addition of ALA and light treatment. Moreover, in comparison to pure MTA, it was shown that the MTA-ALA combination had similar DTS values, XRD patterns, and induction ability of hydroxyapatite mineralization after SBF immersion. MTA has antibacterial properties against *S. aureus* and *P. aeruginosa* [42,43]. The genotoxic effect of 5-ALA is responsible for its antibacterial properties. When light-treated ALA was added to MTA, the inhibition zones against *S. aureus* and *P. aeruginosa* were larger, compared with pure MTA. Moreover, the inhibition zones were also larger than the control (Ca(OH)_2_), which was already known to be an effective bactericidal agent [44,45]. To further verify this point, the relative amounts of *S. aureus* and *P. aeruginosa* attached on Ca(OH)_2_, MH, and MA, with or without light treatment, were evaluated. The results were similar. MTA in combination with light-treated ALA had a lower bacterial count. This ALA based photodynamic antimicrobial strategy can be applied on MTA, and truly improve the properties and antibacterial ability of MTA for root canal sealing treatment.

MTA-ALA with light treatment not only has improved antibacterial ability, it also has better cytocompatibility. Cell viability of hDPCs cultured on MTA-ALA with light treatment at days 1 and 7, were higher than other groups, and they displayed well-defined morphology. In this study, percentages of different cell cycle phases of hDPCs were also evaluated. Higher percentages of cells were found to exist in G2/M stages for all MTA groups, compared to the control groups. Light treated MTA-ALA, also showed a higher percentage of cells existing in the S phase, compared to the rest of the groups. Previous studies showed that MTA induced a small increase in S and G2 phases of the cell cycle, which also implied that MTA will induce proliferation, but not apoptosis, of pulp cells in vitro [46]. In addition, it was also found that cells in S and G2 stages, when exposed to light and ALA, could lead to increased levels of PpIX [47]. Furthermore, as there is no heat involved in the whole treatment process, integrity of extracellular matrix components (i.e., collagen and elastin) can be preserved, and at the same time protect the integrity of cells [48].

Other studies had also shown that MTA is an excellent pulp capping material because of its osteoinductive and osteoconductive properties [49,50]. In this study, effects of MTA in combination with photodynamic antibacterial ALA on odontogenic differentiation of cells were evaluated. The results showed that expression levels of DMP-1, DSPP, ALP, and OC, the odontogenic biomarkers of hDPCs cultured on MTA, were higher than Ctl and CtlA without light treatment. This is consistent with other previous studies [14]. Moreover, the highest expression levels of odontoblastic differentiation markers were observed in the MTA-ALA with light treatment group. It is also possible that there is a correlation between percentage of cells remaining in S phase, and upregulation of S phase associated differentiation genes (i.e., DNA metabolic processes and DNA replication) [51]. Moreover, the results of alizarin red S also implied that MTA-ALA with light treatment can increase the calcium nodule in cell formation. Our promising results validated that the ALA-based photodynamic antibacterial strategy applied on MTA, could facilitate odontoblastic differentiation of hDPCs.

## 5. Conclusions

Even though MTA is an existing excellent pulp capping material, addition of photodynamic antibacterial ALA to MTA showed to have greater antibacterial ability, cytocompatibility, and odontoblastic differentiation potential, compared to MTA alone. Therefore, this is a promising strategy, which can improve root canal treatment, and there is potential for its application in future clinical practice.
